# Development and External Validation of Clinical Features-based Machine Learning Models for Predicting COVID-19 in the Emergency Department

**DOI:** 10.5811/westjem.60243

**Published:** 2023-12-22

**Authors:** Joyce Tay, Yi-Hsuan Yen, Kevin Rivera, Eric H Chou, Chih-Hung Wang, Fan-Ya Chou, Jen-Tang Sun, Shih-Tsung Han, Tzu-Ping Tsai, Yen-Chia Chen, Toral Bhakta, Chu-Lin Tsai, Tsung-Chien Lu, Matthew Huei-Ming Ma

**Affiliations:** * National Taiwan University Hospital, Department of Emergency Medicine, Taipei, Taiwan; † Baylor Scott and White All Saints Medical Center, Department of Emergency Medicine, Fort Worth, Texas; ‡ Texas Christian University, School of Medicine, Fort Worth, Texas; § Baylor University Medical Center, Department of Emergency Medicine, Dallas, Texas; ∥ National Taiwan University, College of Medicine, Department of Emergency Medicine, Taipei, Taiwan; ¶ Far Eastern Memorial Hospital, Department of Emergency Medicine, New Taipei City, Taiwan; # Chang Gung Memorial Hospital at Linkou, Department of Emergency Medicine, Taoyuan, Taiwan; ** Taipei Veterans General Hospital, Department of Emergency Medicine, Taipei, Taiwan; †† National Taiwan University Hospital Yunlin Branch, Department of Emergency Medicine, Yunlin County, Taiwan

## Abstract

**Introduction:**

Timely diagnosis of patients affected by an emerging infectious disease plays a crucial role in treating patients and avoiding disease spread. In prior research, we developed an approach by using machine learning (ML) algorithms to predict serious acute respiratory syndrome coronavirus 2 (SARS-CoV-2) infection based on clinical features of patients visiting an emergency department (ED) during the early coronavirus 2019 (COVID-19) pandemic. In this study, we aimed to externally validate this approach within a distinct ED population.

**Methods:**

To create our training/validation cohort (model development) we collected data retrospectively from suspected COVID-19 patients at a US ED from February 23–May 12, 2020. Another dataset was collected as an external validation (testing) cohort from an ED in another country from May 12–June 15, 2021. Clinical features including patient demographics and triage information were used to train and test the models. The primary outcome was the confirmed diagnosis of COVID-19, defined as a positive reverse transcription polymerase chain reaction test result for SARS-CoV-2. We employed three different ML algorithms, including gradient boosting, random forest, and extra trees classifiers, to construct the predictive model. The predictive performances were evaluated with the area under the receiver operating characteristic curve (AUC) in the testing cohort.

**Results:**

In total, 580 and 946 ED patients were included in the training and testing cohorts, respectively. Of them, 98 (16.9%) and 180 (19.0%) were diagnosed with COVID-19. All the constructed ML models showed acceptable discrimination, as indicated by the AUC. Among them, random forest (0.785, 95% confidence interval [CI] 0.747–0.822) performed better than gradient boosting (0.774, 95% CI 0.739–0.811) and extra trees classifier (0.72, 95% CI 0.677–0.762). There was no significant difference between the constructed models.

**Conclusion:**

Our study validates the use of ML for predicting COVID-19 in the ED and demonstrates its potential for predicting emerging infectious diseases based on models built by clinical features with temporal and spatial heterogeneity. This approach holds promise for scenarios where effective diagnostic tools for an emerging infectious disease may be lacking in the future.

Population Health Research Capsule What do we already know about this issue?

*Timely diagnosis of an emerging infectious disease like COVID-19 is crucial for treatment and prevention.*

What was the research question?

*Can machine learning models predict COVID-19 based on features collected from different emergency departments?*

What was the major finding of the study?

*Random forest achieved good performance (AUC 0.785, 95% CI 0.747–0.822) for COVID-19 prediction.*

How does this improve population health?

*Machine learning can quickly predict COVID-19 in diverse EDs, holding promise for early diagnosis and control of emerging infectious diseases.*

## INTRODUCTION

The global impact of the coronavirus 2019 (COVID-19) pandemic, caused by the severe acute respiratory syndrome coronavirus 2 (SARS-CoV-2), has been far reaching.^1^ ^,^ ^2^ Its clinical manifestations vary from mild to severe illness and even death, with a subset of those infected remaining asymptomatic.^3^ The worldwide crisis has resulted in a significant loss of life and deeply affected global health. Effectively controlling disease transmission requires early recognition and quarantine measures; however, this was difficult before the identification of the causal pathogen and the advent of the molecular diagnostic tool during the early phase of the pandemic.

Taiwan had success in preventing COVID-19 outbreaks until mid-May 2021 when community transmission emerged and cases surged to over 3,100 in a week.^4^ As of September 20, 2022, Taiwan has reported over six million cases and over 5,000 deaths. The sudden surge in cases, coupled with shortages of vaccine and testing, triggered a surge of patients seeking care in the emergency department (ED). This surge significantly impacted healthcare professionals, rendering them susceptible to burnout and emotional strain.^5^ ^–^ ^8^ Tools to reduce workload and streamline processes for healthcare personnel are crucial to ease their mental health burden during a pandemic.

When facing an emerging infectious disease such as COVID-19, it is crucial to identify patients with the risk of infection and thus avoid spreading the disease into the community. For timely recognition of COVID-19 cases, various machine learning (ML) models were developed using a combination of clinical and laboratory reports,^9^ ^–^ ^12^ with some requiring imaging data.^13^ ^–^ ^15^ However, such data may not be readily available during ED triage, hindering early risk stratification. Moreover, any additional diagnostic tests further pose risk to healthcare personnel and require transport and movement of the patient, which should be minimized from an infection prevention and control perspective.^16^ Hence, a persistent challenge remained: how to provide an accurate prediction of SARS-CoV-2 infection in suspected patients with limited modalities of data.

By employing clinical features ascertained during initial ED triage, we previously constructed ML models to create a preliminary screening mechanism that would effectively identify individuals with SARS-CoV-2 infection.^17^ Based on the framework established in that earlier study, we sought external validation of our proposed methodology in the setting of an ED in a tertiary medical facility in Taiwan. Of note, this ED consists of a distinctive population of patients with dissimilar demographic characteristics (in contrast to the cohort used for the original model development). Our primary goal was to validate the feasibility of our approach, to expedite the process of risk stratification pertinent to emerging infectious diseases within the ED.

## METHODS

### Study Design and Setting

We previously conducted a retrospective cohort study by retrieving electronic health record (EHR) data of suspected COVID-19 patients from February 23–May 12, 2020 at the ED of Baylor Scott & White All Saints Medical Center (BAS) in Fort Worth, TX, a 574-bed, university-affiliated tertiary teaching hospital with ≈50,000 ED visits annually. In the current study, we retrospectively collected another set of patient records from suspected adult COVID-19 cases from 12 May 12–June 15, 2021 at the ED of National Taiwan University Hospital (NTUH), Taipei in Taiwan, a 2,400-bed university-affiliated tertiary teaching hospital with a daily census of ≈8,000 outpatients and 300 emergency visits. This study was approved by the Baylor Scott & White Research Institute Insitutional Review Board (No.: 344143), and by NTUH (No. 202009106RIPA), which waived the requirement for informed consent.

### Study Population

In the retrospective study that served as the model development cohort, we identified all patients who presented at the ED of the study hospital with suspected COVID-19 and underwent testing for SARS-CoV-2 through the reverse transcription polymerase chain reaction (RT-PCR) method. In the current study, we also retrospectively collected clinical data for all adult (≥18 years) patients who were tested for SARS-CoV-2 using RT-PCR for suspected COVID-19 as the model’s external validation cohort. The decision to perform RT-PCR tests was left to the discretion of the emergency physician or physician assistant of each patient.

### Data Collection and Outcome Measures

Patient demographics, past medical histories (PMH), vital signs recorded at ED triage, and presenting symptoms were retrieved from the EHR. The comprehensive process of data collection was elaborated in our previous study.^17^ A positive RT-PCR for SARS-CoV-2 confirms the diagnosis of COVID-19 (or SARS-CoV-2 infection) and was defined as the primary outcome in both cohorts. We used the model development cohort as the training/validation set to construct the ML models, and the external validation cohort was used as the testing set to evaluate the models’ performance.

Data were entered, processed, and analyzed with SPSS Statistics for Windows version 27.0, (IBM Corp, Armonk, NY). We performed the assessment of data normality using the Shapiro-Wilk test for continuous variables. The results were subsequently reported as either the mean with standard deviation or the median with interquartile range. Categorical variables were denoted as proportions or percentages. To identify pertinent features, we used univariate analyses to discern disparities in outcomes among distinct groups. These analyses encompassed statistical methods such as the Student *t*-test, chi-squared test, Fisher exact test, or Mann-Whitney U test depending on the distribution. We subsequently selected variables with *P* < 0.1 on the training/validation set as the input features for the development of the ML models. We used K-fold cross-validation to train the model by setting k from 7 to 10, and the selection of k was based on the best area under the receiver operating characteristic curve (AUC) performance on the test set.

In our preceding study, we employed three distinct ML algorithms—specifically, gradient boosting, random forest, and extra trees classifiers—to construct prediction models for forecasting SARS-CoV-2 infection.^17^ In the current study, we validated this approach in another ED population, wherein we replicated the predictive modeling methodology through the employment of the identical ML algorithms used in our prior research. These ML algorithms represent sophisticated ensemble techniques that amalgamate multiple individual models to enhance predictive accuracy and robustness for classification tasks. To deal with the intricate challenge posed by imbalanced data within our cohorts, we applied the synthetic minority oversampling technique (SMOTE), after technique to oversample the minority class, augmenting it by a factor of 0.6 times relative to the magnitude of the majority class. We undertook this measure to establish a more balanced representation, so that the ratio of COVID-19 positive to negative was 0.6 to 1.0 during the training phase. Subsequently, we assessed the performance metrics exhibited by the developed ML models used in the testing set.

To evaluate the performance of the models we built, we used different performance metrics, including the area under the receiver operating characteristic curve (AUC), accuracy, F1-score, precision (positive predictive value [PPV], recall (sensitivity), specificity, negative predictive value (NPV), and area under the precision-recall curve (AUPRC). We used the DeLong test for AUC and Boyd test for AUPRC for pairwise comparisons of the models’ performances. All ML analyses were performed using Jupyter Notebook 6.0.3 (Project Jupyter) with Python 3.8.3 installed and the package scikit-learn 0.23.1 (Python Software Foundation).

## RESULTS

The model development cohort (training/validation set) consisted of 580 cases from patients who presented to BAS, while the model validation cohort (testing set) comprised 946 cases from patients who presented to NTUH. Among them, 98 (16.9%) and 180 (19.0%), respectively, were diagnosed with COVID-19. The characteristics of the study population are shown in Table 1. The characteristics and univariate analyses of variables (features) between patients with COVID-19 are summarized in Table 2, for the training/validation and testing sets, respectively.

**Table 1.** Characteristics of the study population.

Variables (features)	Total (n = 1,526)	Training cohort (n = 580)	Testing cohort (n = 946)	*P* value
Demographics				
Age (years), mean (SD)	52.6 (19.4)	53.7 (18.9)	51.9 (19.6)	0.09
Gender				<0.001^**^
Male	670 (43.9)	213 (36.7)	457 (48.3)	
Female	856 (56.1)	367 (63.3)	489 (51.7)	
EMS transport	359 (23.5)	151 (26.0)	208 (22.0)	<0.001^**^
Triage				<0.001^**^
1	131 (8.6)	5 (0.9)	126 (13.3)	
2	315 (20.6)	149 (25.7)	166 (17.5)	
3	865 (56.7)	416 (71.7)	449 (47.5)	
4	140 (9.2)	9 (1.6)	131 (13.8)	
5	75 (4.9)	1 (0.2)	74 (7.8)	
Temperature, mean (SD)	37.3 (0.8)	37.2 (0.7)	37.4 (0.9)	<0.001^**^
Pulse rate, mean (SD)	96.6 (21.2)	92.8 (20.3)	99.0 (21.4)	<0.001^**^
Respiratory rate, mean (SD)	19.9 (4.8)	18.8 (3.6)	20.5 (5.3)	<0.001^**^
SBP, mean (SD)	132.6 (26.4)	137.9 (25.7)	129.3 (26.4)	<0.001^**^
DBP, mean (SD)	79.4 (16.4)	80.2 (16.7)	78.9 (16.2)	0.12
SpO_2_, mean (SD)	96.7 (4.1)	97.4 (3.4)	96.3 (4.5)	<0.001^**^
Oxygen therapy	199 (13.0)	70 (12.1)	129 (13.6)	0.5
Weight, mean (SD)	73.1 (23.7)	88.9 (26.1)	63.4 (15.6)	<0.001^**^
Height, mean (SD)	1.7 (0.4)	1.7 (0.1)	1.7 (0.5)	0.16
BMI, mean (SD)	26.6 (7.8)	31.5 (9.0)	23.5 (4.9)	<0.001^**^
Smoking history				<0.001^**^
Yes	297 (19.5)	187 (32.2)	110 (11.6)	
No	773 (50.7)	376 (64.8)	397 (42)	
Unknown	456 (29.9)	17 (2.9)	439 (46.4)	
Travel history	348 (22.8)	36 (6.2)	312 (33.0)	<0.001^**^
Contact history	329 (21.6)	110 (19.0)	219 (23.2)	<0.001^**^
Duration, days, mean (SD)	4.1 (6)	5.7 (7.7)	3.1 (4.5)	<0.001^**^
AMS	123 (8.1)	30 (5.2)	93 (9.8)	0.001^**^
Seizures	15 (1.0)	4 (0.7)	11 (1.2)	0.36
Fever	673 (44.1)	266 (45.9)	407 (43.0)	0.42
Chills	130 (8.5)	84 (14.5)	46 (4.9)	<0.001^**^
Myalgia	218 (14.3)	131 (22.6)	87 (9.2)	<0.001^**^
Arthralgia	18 (1.2)	11 (1.9)	7 (0.7)	0.04^**^
Headache	199 (13.0)	116 (20.0)	83 (8.8)	<0.001^**^
Facial pain	9 (0.6)	4 (0.7)	5 (0.5)	0.69
Red eyes	6 (0.4)	5 (0.9)	1 (0.1)	0.02^**^
Otalgia	16 (1.0)	10 (1.7)	6 (0.6)	0.04^**^
Sore throat	332 (21.8)	81 (14.0)	251 (26.5)	<0.001^**^
Rhinorrhea	172 (11.3)	26 (4.5)	146 (15.4)	<0.001^**^
Stuffy nose	92 (6.0)	69 (11.9)	23 (2.4)	<0.001^**^
Sneezing	12 (0.8)	8 (1.4)	4 (0.4)	0.04^**^
Postnasal drip	7 (0.5)	5 (0.9)	2 (0.2)	0.07
Hypogeusia/ageusia	14 (0.9)	3 (0.5)	11 (1.2)	0.2
hyposmia/anosmia	14 (0.9)	6 (1.0)	8 (0.8)	0.71
Hoarseness	6 (0.4)	1 (0.2)	5 (0.5)	0.28
Dysphagia	23 (1.5)	6 (1.0)	17 (1.8)	0.24
Cough	715 (46.9)	362 (62.4)	353 (37.3)	<0.001^**^
Sputum	197 (12.9)	47 (8.1)	150 (15.9)	<0.001^**^
SOB	535 (35.1)	334 (57.6)	201 (21.2)	<0.001^**^
Malaise	240 (15.7)	110 (19.0)	130 (13.7)	0.007^**^
Diarrhea	202 (13.2)	65 (11.2)	137 (14.5)	0.07
Vomiting	126 (8.3)	66 (11.4)	60 (6.3)	<0.001^**^
Nausea	154 (10.1)	115 (19.8)	39 (4.1)	<0.001^**^
Anorexia	52 (3.4)	26 (4.5)	26 (2.7)	0.07
Abdominal pain	132 (8.7)	62 (10.7)	70 (7.4)	0.03^**^
Chest pain	190 (12.5)	120 (20.7)	70 (7.4)	<0.001^**^
Hemoptysis	12 (0.8)	6 (1.0)	6 (0.6)	0.39
Skin lesion	9 (0.6)	5 (0.9)	4 (0.4)	0.28
Skin itch	9 (0.6)	3 (0.5)	6 (0.6)	0.77
Paresthesia	7 (0.5)	3 (0.5)	4 (0.4)	0.79
Back pain	51 (3.3)	38 (6.6)	13 (1.4)	<0.001^**^
Neuropathy	1 (0.1)	1 (0.2)	0 (0.0)	0.2
Renal colic/flank pain	18 (1.2)	15 (2.6)	3 (0.3)	<0.001^**^
Cormorbidities (if any)	906 (59.4)	450 (77.6)	456 (48.2)	<0.001^**^
Cormorbidities (>1)	646 (42.3)	355 (61.2)	291 (30.8)	<0.001^**^
COPD	87 (5.7)	66 (11.4)	21 (2.2)	<0.001^**^
Asthma	132 (8.7)	99 (17.1)	33 (3.5)	<0.001^**^
DM	281 (18.4)	149 (25.7)	132 (14.0)	<0.001^**^
HTN	497 (32.6)	276 (47.6)	221 (23.4)	<0.001^**^
CAD	115 (7.5)	55 (9.5)	60 (6.3)	0.02^**^
CHF	81 (5.3)	52 (9.0)	29 (3.1)	<0.001^**^
CVA	77 (5.0)	36 (6.2)	41 (4.3)	0.1
Hepatitis B	28 (1.8)	0 (0.0)	28 (3.0)	<0.001^**^
Hepatitis C	15 (1.0)	11 (1.9)	4 (0.4)	0.005^**^
Cirrhosis	20 (1.3)	14 (2.4)	6 (0.6)	0.003^**^
Cancer	213 (14.0)	74 (12.8)	139 (14.7)	0.29
Current chemotherapy	56 (3.7)	11 (1.9)	45 (4.8)	0.004^**^
CKD	102 (6.7)	76 (13.1)	26 (2.7)	<0.001^**^
ESRD	49 (3.2)	32 (5.5)	17 (1.8)	<0.001^**^
History of solid organ transplant	24 (1.6)	18 (3.1)	6 (0.6)	<0.001^**^
Immunodeficiency	132 (8.7)	5 (0.9)	127 (13.4)	<0.001^**^
HIV	11 (0.7)	4 (0.7)	7 (0.7)	0.91
Rheumatologic diseases	34 (2.2)	17 (2.9)	17 (1.8)	0.15
Dementia	24 (1.6)	11 (1.9)	13 (1.4)	0.43
PUD	21 (1.4)	1 (0.2)	20 (2.1)	0.002^**^
Gastroparesis	5 (0.3)	4 (0.7)	1 (0.1)	0.05
Migraine	19 (1.2)	17 (2.9)	2 (0.2)	<0.001^**^
Fibromyalgia	8 (0.5)	4 (0.7)	4 (0.4)	0.48
Chronic pain syndrome	31 (2.0)	24 (4.1)	7 (0.7)	<0.001^**^
Alcohol use disorder	9 (0.6)	5 (0.9)	4 (0.4)	0.28
Substance use disorder	27 (1.8)	24 (4.1)	3 (0.3)	<0.001^**^
Depression	70 (4.6)	55 (9.5)	15 (1.6)	<0.001^**^
Psychiatric disease	73 (4.8)	52 (9.0)	21 (2.2)	<0.001^**^
Pregnancy	21 (1.4)	19 (3.3)	2 (0.2)	<0.001^**^

*EMS*, emergency medical services; *SBP,* systolic blood pressure; *DBP*, diastolic blood pressure; SpO_2_, oxygen saturation; *BMI*, body mass index; *AMS*, altered mental status; *SOB*, shortness of breath; *COPD*, chronic obstruction pulmonary disease; *DM*, diabetes mellitus; *HTN*, hypertension; *CAD*, coronary artery disease; *CHF*, congestive heart failure; *CVA*, cerebrovascular accident; *CKD*, chronic kidney disease; *ESRD*, end stage renal disease; *PUD*, peptic ulcer disease.

Note: ^**^ *P* < 0.05.

**Table 2.** Characteristics and univariate analyses of variables (features) between patients with or without COVID-19 on the training and testing cohorts.

	Training cohort (n = 580)		*P* value	Testing cohort (n = 946)		*P* value
	COVID-19 (−) (n = 482)	COVID-19 (+) (n = 98)		COVID-19 (−) (n = 766)	COVID-19 (+) (n = 180)	
Demographics						
Age (y), mean (SD)	54.4 (18.9)	50.3 (18.7)	0.05^*^	50.3 (20.3)	58.9 (14.6)	<0.001^*^
Gender			0.36			0.24
Female	309 (64.1)	58 (59.2)		403 (52.6)	86 (47.8)	
Male	173 (35.9)	40 (40.8)		363 (47.4)	94 (52.2)	
EMS transport	132 (27.4)	19 (19.4)	0.1	144 (18.8)	64 (35.6)	<0.001^*^
Triage			0.43			0.07^*^
1	3 (0.6)	2 (2.0)		98 (12.8)	28 (15.6)	
2	129 (26.8)	20 (20.4)		139 (18.1)	27 (15.0)	
3	342 (71.0)	74 (75.5)		352 (46.0)	97 (53.9)	
4	7 (1.5)	2 (2.0)		116 (15.1)	15 (8.3)	
5	1 (0.2)	0 (0.0)		61 (8.0)	13 (7.2)	
Temperature, mean (SD)	37.2 (0.7)	37.6 (0.7)	<0.001^*^	37.3 (0.8)	37.9 (0.9)	<0.001^*^
Pulse rate, mean (SD)	92.5 (20.7)	94.1 (18.1)	0.46	98.9 (22.2)	99.4 (18.0)	0.77
Respiratory rate, mean (SD)	18.7 (3.4)	19.5 (4.4)	0.05^*^	20.3 (5.4)	21.5 (4.9)	0.007^*^
SBP, mean (SD)	139.0 (26.2)	133.0 (22.2)	0.04^*^	128.9 (26.8)	130.7 (24.3)	0.42
DBP, mean (SD)	80.2 (17.2)	80.0 (13.7)	0.9	78.9 (16.7)	78.7 (14.1)	0.88
SpO_2_, mean (SD)	97.6 (3.1)	96.3 (4.3)	<0.001^*^	96.6 (3.9)	94.9 (6.3)	<0.001^*^
Oxygen therapy	59 (12.2)	11 (11.2)	0.78	90 (11.7)	39 (21.7)	<0.001^*^
Weight, mean (SD)	87.6 (26.1)	95.0 (25.6)	0.01^*^	63.4 (16.3)	63.7 (12.5)	0.81
Height, mean (SD)	1.7 (0.1)	1.7 (0.1)	0.53	1.7 (0.6)	1.6 (0.1)	0.51
BMI, mean (SD)	31.1 (9.1)	33.7 (8.4)	0.009^*^	23.4 (5.1)	23.8 (3.8)	0.3
Smoking history			<0.001^*^			<0.001^*^
Yes	169 (35.1)	18 (18.4)		80 (10.4)	30 (16.7)	
No	305 (63.3)	71 (72.4)		276 (36)	121 (67.2)	
Unknown	8 (1.7)	9 (9.2)		410 (53.5)	29 (16.1)	
Travel history	23 (4.8)	13 (13.3)	0.001^*^	204 (26.6)	108 (60.0)	<0.001^*^
Contact history	59 (12.2)	51 (52.0)	<0.001^*^	112 (14.6)	107 (59.4)	<0.001^*^
Duration, days, mean (SD)	5.9 (8.2)	5.1 (3.7)	0.35	2.9 (4.3)	3.7 (5)	0.04^*^
AMS	29 (6.0)	1 (1.0)	0.04^*^	82 (10.7)	11 (6.1)	0.06^*^
Seizures	4 (0.8)	0 (0.0)	0.37	10 (1.3)	1 (0.6)	0.4
Fever	197 (40.9)	69 (70.4)	<0.001^*^	287 (37.5)	120 (66.7)	<0.001^*^
Chills	72 (14.9)	12 (12.2)	0.49	36 (4.7)	10 (5.6)	0.63
Myalgia	98 (20.3)	33 (33.7)	0.004^*^	60 (7.8)	27 (15.0)	0.003^*^
Arthralgia	11 (2.3)	0 (0.0)	0.13	6 (0.8)	1 (0.6)	0.75
Headache	94 (19.5)	22 (22.4)	0.51	72 (9.4)	11 (6.1)	0.16
Facial pain	4 (0.8)	0 (0.0)	0.37	5 (0.7)	0 (0.0)	0.28
Red eyes	5 (1.0)	0 (0.0)	0.31	0 (0.0)	1 (0.6)	0.04^*^
Otalgia	8 (1.7)	2 (2.0)	0.79	6 (0.8)	0 (0.0)	0.23
Sore throat	70 (14.5)	11 (11.2)	0.39	205 (26.8)	46 (25.6)	0.74
Rhinorrhea	19 (3.9)	7 (7.1)	0.16	128 (16.7)	18 (10.0)	0.02^*^
Stuffy nose	55 (11.4)	14 (14.3)	0.42	21 (2.7)	2 (1.1)	0.2
Sneezing	7 (1.5)	1 (1.0)	0.74	4 (0.5)	0 (0.0)	0.33
Postnasal drip	4 (0.8)	1 (1.0)	0.85	2 (0.3)	0 (0.0)	0.49
Hypogeusia/ageusia	0 (0.0)	3 (3.1)	<0.001^*^	6 (0.8)	5 (2.8)	0.02^*^
hyposmia/anosmia	3 (0.6)	3 (3.1)	0.03^*^	6 (0.8)	2 (1.1)	0.67
Hoarseness	1 (0.2)	0 (0.0)	0.65	3 (0.4)	2 (1.1)	0.23
Dysphagia	6 (1.2)	0 (0.0)	0.27	16 (2.1)	1 (0.6)	0.16
Cough	285 (59.1)	77 (78.6)	<0.001^*^	248 (32.4)	105 (58.3)	<0.001^*^
Sputum	35 (7.3)	12 (12.2)	0.1	114 (14.9)	36 (20.0)	0.09^*^
SOB	277 (57.5)	57 (58.2)	0.9	141 (18.4)	60 (33.3)	<0.001^*^
Malaise	90 (18.7)	20 (20.4)	0.69	98 (12.8)	32 (17.8)	0.08^*^
Diarrhea	48 (10.0)	17 (17.3)	0.03^*^	114 (14.9)	23 (12.8)	0.47
Vomiting	57 (11.8)	9 (9.2)	0.45	57 (7.4)	3 (1.7)	0.004^*^
Nausea	92 (19.1)	23 (23.5)	0.32	36 (4.7)	3 (1.7)	0.07^*^
Anorexia	21 (4.4)	5 (5.1)	0.75	19 (2.5)	7 (3.9)	0.3
Abdominal pain	54 (11.2)	8 (8.2)	0.37	60 (7.8)	10 (5.6)	0.29
Chest pain	106 (22.0)	14 (14.3)	0.09^*^	55 (7.2)	15 (8.3)	0.59
Hemoptysis	4 (0.8)	2 (2.0)	0.28	4 (0.5)	2 (1.1)	0.37
Skin lesion	5 (1.0)	0 (0.0)	0.31	2 (0.3)	2 (1.1)	0.11
Skin itch	3 (0.6)	0 (0.0)	0.43	6 (0.8)	0 (0.0)	0.23
Paresthesia	2 (0.4)	1 (1.0)	0.45	4 (0.5)	0 (0.0)	0.33
Back pain	33 (6.8)	5 (5.1)	0.52	11 (1.4)	2 (1.1)	0.74
Neuropathy	0 (0.0)	1 (1.0)	0.03^*^	0 (0.0)	0 (0.0)	NA
Renal colic/flank pain	12 (2.5)	3 (3.1)	0.75	3 (0.4)	0 (0.0)	0.4
Comorbidities (if any)	389 (80.7)	61 (62.2)	<0.001^*^	365 (47.7)	91 (50.6)	0.48
Comorbidities (>1)	304 (63.1)	51 (52.0)	0.04^*^	245 (32.0)	46 (25.6)	0.09
COPD	63 (13.1)	3 (3.1)	0.004^*^	18 (2.3)	3 (1.7)	0.58
Asthma	86 (17.8)	13 (13.3)	0.27	29 (3.8)	4 (2.2)	0.3
DM	125 (25.9)	24 (24.5)	0.77	101 (13.2)	31 (17.2)	0.16
HTN	236 (49.0)	40 (40.8)	0.14	170 (22.2)	51 (28.3)	0.08^*^
CAD	46 (9.5)	9 (9.2)	0.91	51 (6.7)	9 (5.0)	0.41
CHF	45 (9.3)	7 (7.1)	0.49	28 (3.7)	1 (0.6)	0.03^*^
CVA	35 (7.3)	1 (1.0)	0.02^*^	35 (4.6)	6 (3.3)	0.46
Hepatitis B	0 (0.0)	0 (0.0)	NA	23 (3.0)	5 (2.8)	0.87
Hepatitis C	10 (2.1)	1 (1.0)	0.49	4 (0.5)	0 (0.0)	0.33
Cirrhosis	14 (2.9)	0 (0.0)	0.09^*^	6 (0.8)	0 (0.0)	0.23
Cancer	66 (13.7)	8 (8.2)	0.13	123 (16.1)	16 (8.9)	0.01^*^
Current chemotherapy	9 (1.9)	2 (2.0)	0.91	43 (5.6)	2 (1.1)	0.01^*^
CKD	63 (13.1)	13 (13.3)	0.96	25 (3.3)	1 (0.6)	0.05^*^
ESRD	29 (6.0)	3 (3.1)	0.24	15 (2.0)	2 (1.1)	0.44
History of solid organ transplant	17 (3.5)	1 (1.0)	0.19	5 (0.7)	1 (0.6)	0.88
Immunodeficiency	5 (1.0)	0 (0.0)	0.31	112 (14.6)	15 (8.3)	0.03^*^
HIV	4 (0.8)	0 (0.0)	0.37	6 (0.8)	1 (0.6)	0.75
Rheumatologic diseases	16 (3.3)	1 (1.0)	0.22	14 (1.8)	3 (1.7)	0.88
Dementia	8 (1.7)	3 (3.1)	0.35	12 (1.6)	1 (0.6)	0.29
PUD	1 (0.2)	0 (0.0)	0.65	15 (2.0)	5 (2.8)	0.49
Gastroparesis	4 (0.8)	0 (0.0)	0.37	1 (0.1)	0 (0.0)	0.63
Migraine	15 (3.1)	2 (2.0)	0.57	2 (0.3)	0 (0.0)	0.49
Fibromyalgia	4 (0.8)	0 (0.0)	0.37	2 (0.3)	2 (1.1)	0.11
Chronic pain syndrome	20 (4.1)	4 (4.1)	0.98	5 (0.7)	2 (1.1)	0.52
Alcohol use disorder	5 (1.0)	0 (0.0)	0.31	3 (0.4)	1 (0.6)	0.76
Substance use disorder	21 (4.4)	3 (3.1)	0.56	3 (0.4)	0 (0.0)	0.4
Depression	54 (11.2)	1 (1.0)	0.002^*^	14 (1.8)	1 (0.6)	0.22
Psychiatric disease	47 (9.8)	5 (5.1)	0.14	17 (2.2)	4 (2.2)	1
Pregnancy	14 (2.9)	5 (5.1)	0.27	2 (0.3)	0 (0.0)	0.49

*EMS*, emergency medical services; *SBP*, systolic blood pressure; *DBP*, diastolic blood pressure; *SpO_2_*, oxygen saturation; *BMI*, body mass index; *AMS*, altered mental status; *SOB*, shortness of breath; *COPD*, chronic obstruction pulmonary disease; *DM*, diabetes mellitus; *HTN*, hypertension; *CAD*, coronary artery disease; *CHF*, congestive heart failure; *CVA*, cerebrovascular accident; *CKD*, chronic kidney disease; *ESRD*, end stage renal disease; *PUD*, peptic ulcer disease.

Note: ^*^ *P* < 0.1.

**Figure 1.**
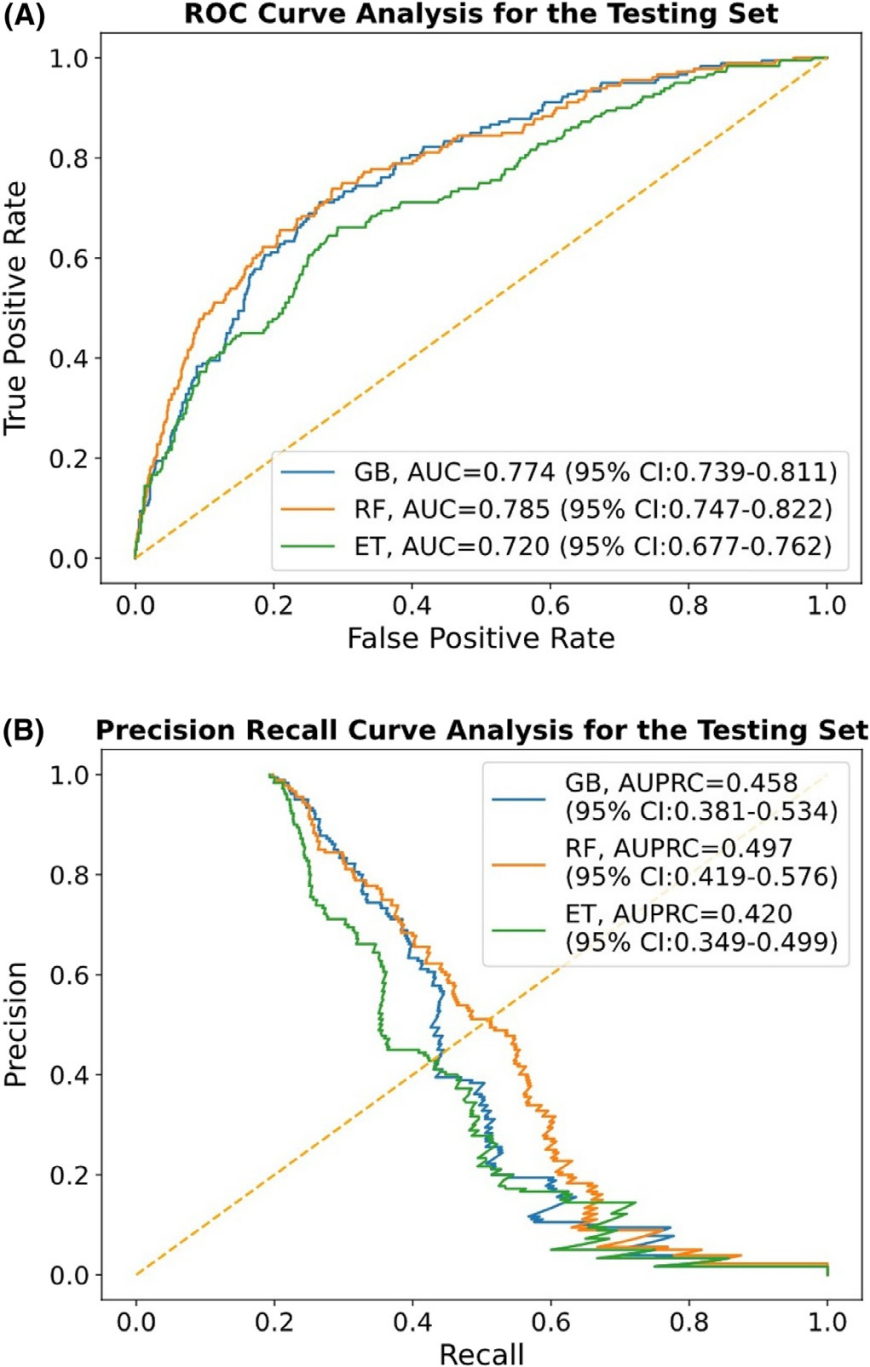
Results of the machine learning models on the test cohort. (A), Receiver operating characteristic (ROC) curves and the comparison of area under curve (AUC); (B), precision-recall curve and the comparison of area under the precision-recall curve (AUPRC) for three different machine learning models. *ET*, extra trees; *RF*, random forest; *GB*, gradient boosting.

We selected 26 features by setting the *P*-value threshold of less than 0.1 from the model development cohort, encompassing six demographics, four triage data, 10 clinical symptoms, and six PMHs. Employing k as 7 for K-fold cross-validation, the classification outcomes for the three different ML models in the testing set are presented in Table 3 and Figure 1. The detailed performance metrics in terms of different k values on the training/validation and testing sets are shown in Supplementary Table 1.

**Table 3.** Performance metrics of 7-fold cross validation for different machine learning algorithms on the testing set.

Models	AUC (95% CI)	AUPRC (95% CI)	Accuracy	F1	Sensitivity	Specificity	PPV	NPV
Gradient boosting	0.774 (0.739–0.811)	0.458 (0.381–0.534)	0.815	0.335	0.244	0.949	0.53	0.842
Random forest	0.785 (0.747–0.822)	0.497 (0.419–0.576)	0.827	0.427	0.339	0.941	0.575	0.858
Extra trees	0.72 (0.677–0.762)	0.42 (0.349–0.499)	0.792	0.426	0.406	0.883	0.448	0.863

*CI*, confidence interval; *AUC*, area under the receiver operating characteristic curve; *AUPRC*, area under the precision recall curve; *PPV*, positive predictive value; *NPV*, negative predictive value.

Among the constructed ML models, random forest demonstrated superior performance with the highest AUC value (0.785, 95% CI 0.747–0.822), followed by gradient boosting (0.774, 95% CI 0.739–0.811) and extra trees classifier (0.720, 95% CI 0.677–0.762). By fine-tuning the tradeoff between precision and recall for different thresholds to calculate the AUPRC, random forest (0.497. 95% CI 0.419–0.576) outperformed gradient boosting (0.458, 95% CI 0.381–0.534) and extra trees classifier (0.420, 95% CI: 0.349–0.499). The differences between each ML model in terms of AUC and AUPRC are not significant.

**Figure 2.**
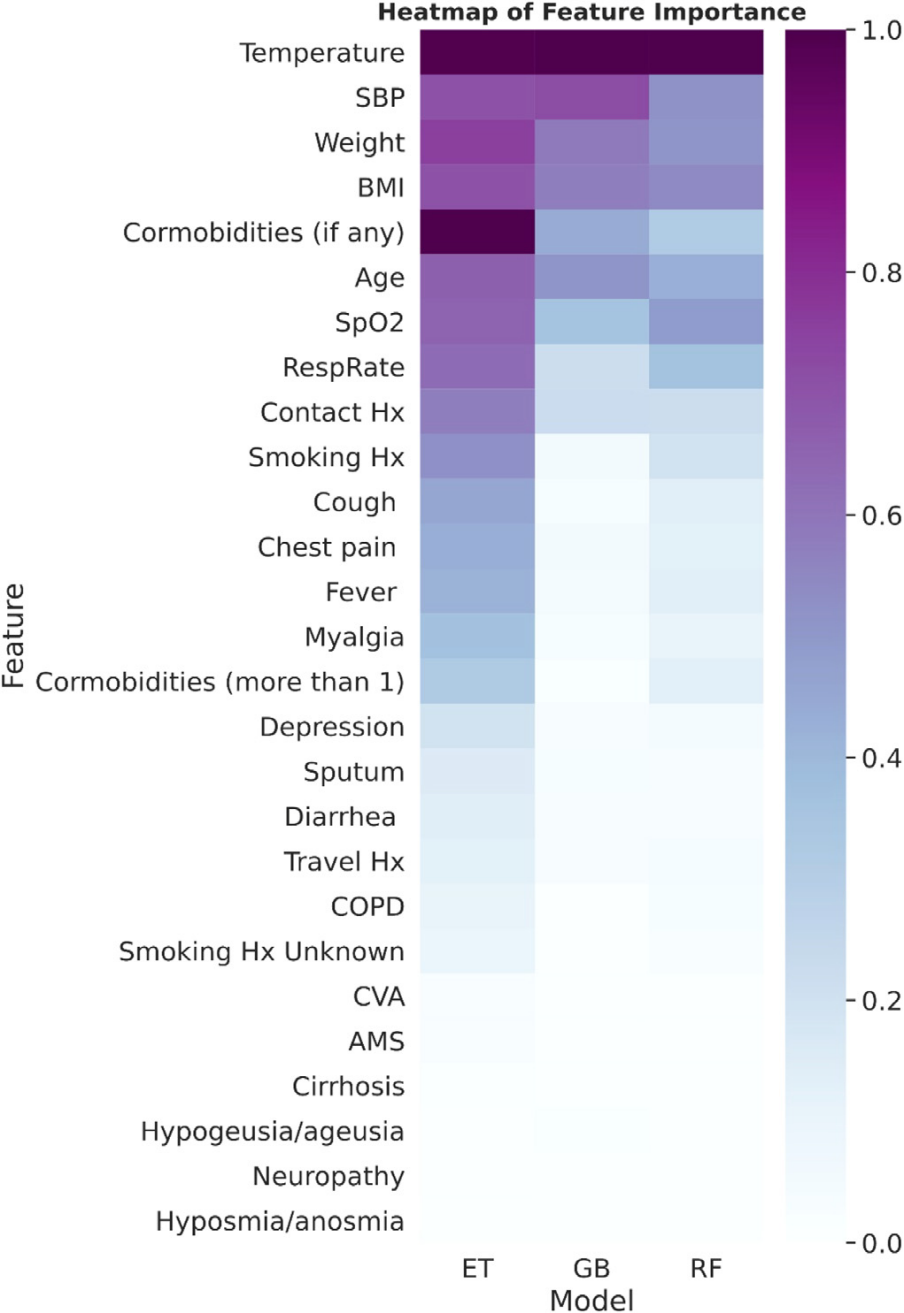
The heat map of computed features ordered by median normalized importance across all models. SBP, systolic blood pressure; *BMI*, body mass index; *SPO_2_*, oxygen saturation; *Hx*, history; *COPD*, chronic obstructive pulmonary disorder; *CVA, cerebrovascular accident;* *AMS*, altered mental state; *ET*, extra trees; *RF*, random forest; *GB*, gradient boosting.

In evaluating additional performance metrics, all our ML models performed well in terms of accuracy, specificity, and NPV. Nevertheless, the performances of the F1 score, sensitivity, and PPV are suboptimal. Feature importance (presented as a heat map computed and ordered by median normalized importance across all models) is shown in Figure 2. The 9 most important features were temperature, systolic blood pressure, weight, body mass index, any co-morbidities, age, oxygen saturation, respiratory rate, and contact history.

## DISCUSSION

### The Main Findings of This Study

In our previous study, we constructed ML models designed to predict COVID-19 based on the clinical features documented during ED triage within a tertiary teaching hospital in the US during the first wave of the COVID-19 pandemic.^17^ In the current study, our objective was to validate this approach externally in another ED population of a medical center located elsewhere in the world. By collecting a cohort of 946 consecutive ED patients visiting NTUH during the second wave of the COVID-19 pandemic in Taiwan, we found that the random forest model emerged as the best performer with acceptable discrimination performance in terms of AUC and AUPRC. However, the remaining two models also achieved close results without significant differences, and all models performed well in accuracy, specificity, and NPV. With only demographics, vital signs at triage, clinical symptoms, contact history and PMH collected at ED triage, this approach exemplifies the feasibility of predicting COVID-19 at triage even before patients go into the ED. The predictive results offer valuable assistance to emergency physicians in identifying patients at risk of the disease. This enables such patients to undergo further examination, testing, isolation, and appropriate treatment measures.

### Comparison with Previous Studies

Since the inception of the disease, ML algorithms have been extensively applied in fighting COVID-19.^18^ While certain applications targeted COVID-19 diagnosis as the primary outcome, others focused on morbidity and mortality for patients with confirmed SARS-CoV-2 infection.^10^ Some investigations focused on the ED setting, while others focused on the general population.^19^ ^,^ ^20^ Moreover, some reports used chest radiographs or computed tomography of the lung to exploit imaging characteristics to differentiate pneumonia caused by SARS-CoV-2 from that with other causes,^13,14,15^ while others used routine blood test results.^9^ ^,^ ^11^ ^,^ ^12^ Meanwhile, certain reports employed clinical data—including patient demographics, symptoms, vital signs, and PMH—as the input of prediction models similar to our study design.^21^ Furthermore, there were studies that combined multiple modalities from the above-mentioned studies.^22^ Although the source and size of the studies reported in the literature varied, our current study is the only one that uses only the clinical features collected from ED triage and provides promising external validation results.

In comparison to this study, our previous study yielded a stronger result with an AUC of 0.86, whereas the best-performing model in this study achieved only an AUC of 0.785. The decline in performance was anticipated since the test dataset in the previous study came from the same population as the training dataset, whereas in this study the two datasets came from different populations with different patient demographics. Additionally, certain features used in our previous study that rely on the model development cohort were not employed in this validation study due to different healthcare systems and ethnicity distribution in different populations. Nonetheless (with the exception of the study by Zoabi et al), the models we built in the current study showed competitive or even better performance in comparison to other studies that relied on clinical features for their models^19^ ^–^ ^22^ (Supplementary Table 2).

### Feasibility for Clinical Application

This study achieved acceptable predictive performances with metrics exceeding 0.7 in terms of AUC, specificity, and NPV, making these ML models a suitable screening tool to rule in patients in need of further attention. With the information readily accessible from the EHR during ED triage, our model may assist emergency clinicians to segregate patients with a high likelihood of COVID-19 infection from those at lower risk. By doing so, the risk of cross-infection may be minimized, and high-risk patients may receive appropriate care promptly. If effectively integrated into the system as an automated alert system during the initial ED encounter, it could exert substantial impact on clinical workflows while simultaneously reduce disease transmission and cross-infection in the ED setting. However, precision must be exercised to ensure the alerts provided by the predictive model are pertinent and timely, without disrupting the existing workflow.^23^

At present, a confirmed diagnosis of COVID-19 is made by direct detection of SARS-CoV-2 RNA using RT–PCR testing; however, it may take up to eight hours to obtain the test result after the sample is delivered.^24^ Although several rapid antigen tests (RAT) have been developed as screening tools, their accuracy is strongly affected by the pretest probability and is less effective in the asymptomatic population.^25^ Moreover, many regions worldwide still lack the capacity for RAT kits. As the COVID-19 pandemic persists and new variants emerge, a reliable ML prediction model could function as a rapid screening tool to quickly differentiate the suspicious cases from other patients and facilitate infection control even before patients enter the ED. Additionally, this study also provides a proof of concept for ML models capable of predicting an emerging infectious disease of an unknown pathogen based on models built by clinical features without the necessity of pathogen-specific tests. When faced with an emerging novel infectious disease in the future, this approach would be extremely valuable, particularly in situations where a dedicated diagnostic tool has yet to be developed or encounters challenges related to supply and demand.

## LIMITATIONS

This study does come with limitations. First, a class imbalance issue was evident. With only 16.9% and 19.0% being diagnosed with COVID-19 in our development and validation cohorts, the diagnostic performances in terms of sensitivity and PPV were suboptimal. However, the performance of AUPRC was acceptable given that the positivity rate of COVID-19 in the testing cohort was only 19%. Second, the study was conducted before widespread vaccination was available in the US (for the training/validation dataset) and in Taiwan (for the test dataset).^17^ and prior to the emergence and dominance of the Omicron variant.^26^ The difference in symptoms could affect the accuracy of the model when the models were trained with cases of different variants of SARS-CoV-2.^27^ However, this approach could be aptly adapted in the future as the model is continuously trained and updated to reflect the new attributes of variant pathogens. It is essential that further prospective studies are undertaken to examine the feasibility of this model being applied to future patients.

## CONCLUSION

Our machine learning approach exhibited acceptable discriminatory performance for screening patients with suspected COVID-19, based on models built in a different patient population characterized by temporal and spatial heterogeneity, and relying solely on clinical features captured during ED triage. This study offers a proof of concept, suggesting the applicability of an ML approach in diagnosing novel emerging infectious diseases within one region by drawing on clinical features collected from another region, especially in circumstances preceding the advent and availability of a rapid diagnostic tool.

## Supplementary Information




