# Supplementary material for: Development and External Validation of Clinical Features-based Machine Learning Models for Predicting COVID-19 in the Emergency Department
Source: West J Emerg Med. 2023 Dec 22;25(1):67–78. doi: 10.5811/westjem.60243 (PMC10777189; doi:10.5811/westjem.60243)
Supplement: Supplementary file 1 [file wjem-25-67-s001.docx]

| **Supplemental Table 1. Performance metrics in terms of different k fold for different machine learning algorithms** | | | | | | | | | | | |
| --- | --- | --- | --- | --- | --- | --- | --- | --- | --- | --- | --- |
| k fold | Cohort | Models | AUC (95% CI) | AUPRC (95% CI) | Accuracy | F1 | Sensitivity | Specificity | PPV | NPV |  |
| 7 | Testing | GradientBoosting | 0.774 (0.739-0.811) | 0.458 (0.381-0.534) | 0.815 | 0.335 | 0.244 | 0.949 | 0.53 | 0.842 |  |
|  |  | RandomForest | 0.785 (0.747-0.822) | 0.497 (0.419-0.576) | 0.827 | 0.427 | 0.339 | 0.941 | 0.575 | 0.858 |  |
|  |  | ExtraTrees | 0.72 (0.677-0.762) | 0.42 (0.349-0.499) | 0.792 | 0.426 | 0.406 | 0.883 | 0.448 | 0.863 |  |
|  | Training | GradientBoosting | 1 (1-1) | 1 (1-1) | 1 | 1 | 1 | 1 | 1 | 1 |  |
|  |  | RandomForest | 1 (1-1) | 1 (1-1) | 0.998 | 0.995 | 0.99 | 1 | 1 | 0.998 |  |
|  |  | ExtraTrees | 1 (1-1) | 1 (1-1) | 1 | 1 | 1 | 1 | 1 | 1 |  |
| 8 | Testing | GradientBoosting | 0.747 (0.708-0.784) | 0.413 (0.34-0.487) | 0.811 | 0.298 | 0.211 | 0.952 | 0.507 | 0.837 |  |
|  |  | RandomForest | 0.783 (0.745-0.82) | 0.493 (0.417-0.575) | 0.829 | 0.426 | 0.333 | 0.945 | 0.588 | 0.858 |  |
|  |  | ExtraTrees | 0.712 (0.669-0.756) | 0.411 (0.341-0.489) | 0.782 | 0.405 | 0.389 | 0.875 | 0.422 | 0.859 |  |
|  | Training | GradientBoosting | 1 (1-1) | 1 (1-1) | 1 | 1 | 1 | 1 | 1 | 1 |  |
|  |  | RandomForest | 1 (1-1) | 1 (1-1) | 1 | 1 | 1 | 1 | 1 | 1 |  |
|  |  | ExtraTrees | 1 (1-1) | 1 (1-1) | 1 | 1 | 1 | 1 | 1 | 1 |  |
| 9 | Testing | GradientBoosting | 0.778 (0.743-0.812) | 0.425 (0.359-0.5) | 0.807 | 0.315 | 0.233 | 0.941 | 0.483 | 0.839 |  |
|  |  | RandomForest | 0.781 (0.742-0.819) | 0.482 (0.408-0.563) | 0.825 | 0.411 | 0.322 | 0.943 | 0.569 | 0.855 |  |
|  |  | ExtraTrees | 0.715 (0.671-0.759) | 0.416 (0.347-0.493) | 0.791 | 0.414 | 0.389 | 0.885 | 0.443 | 0.86 |  |
|  | Training | GradientBoosting | 1 (1-1) | 1 (1-1) | 1 | 1 | 1 | 1 | 1 | 1 |  |
|  |  | RandomForest | 1 (1-1) | 1 (1-1) | 0.998 | 0.995 | 0.99 | 1 | 1 | 0.998 |  |
|  |  | ExtraTrees | 1 (1-1) | 1 (1-1) | 1 | 1 | 1 | 1 | 1 | 1 |  |
| 10 | Testing | GradientBoosting | 0.747 (0.709-0.784) | 0.421 (0.349-0.495) | 0.809 | 0.296 | 0.211 | 0.949 | 0.494 | 0.837 |  |
|  |  | RandomForest | 0.784 (0.745-0.821) | 0.489 (0.412-0.572) | 0.833 | 0.444 | 0.35 | 0.946 | 0.606 | 0.861 |  |
|  |  | ExtraTrees | 0.708 (0.665-0.752) | 0.409 (0.338-0.487) | 0.78 | 0.392 | 0.372 | 0.876 | 0.414 | 0.856 |  |
|  | Training | GradientBoosting | 1 (1-1) | 1 (1-1) | 1 | 1 | 1 | 1 | 1 | 1 |  |
|  |  | RandomForest | 1 (1-1) | 1 (1-1) | 0.998 | 0.995 | 0.99 | 1 | 1 | 0.998 |  |
|  |  | ExtraTrees | 1 (1-1) | 1 (1-1) | 1 | 1 | 1 | 1 | 1 | 1 |  |
|  |  |  |  |  |  |  |  |  |  |  |  |

| **Supplementary Table 2**. Other machine learning studies with models based only on clinical features | | | | | | |  |
| --- | --- | --- | --- | --- | --- | --- | --- |
| **Author & Year [reference No.]** | **Present Study** | **XXX, 2020 [17] (Our previous study)** | **Mei, 2020 [22]** | **Tostmann, 2020 [21]** | **Zoabi, 2021 [19]** | **Martinez-Velazquez, 2021 [20]** |  |
| **Cohort** | ED patients | ED patients | Unspecified (outpatient? ED?) | Healthcare workers | Community | Community |  |
| **Test Set Source** | XXX, XXX | XXX, US | Multicenter (18), China | Radboud University Medical Centre, the Netherlands | Publicly released data (Israeli Ministry of Health) | Sornora and Tlaxcala state health authorities, Mexico |  |
| **Training/Validation Set Size** | 580 | 348 | 626 | 627 | 51831 | 1772 |  |
| **COVID Positive (Training/Validation Set)** | 16.9% | 20.1% | 45.5% | 8.9% | 9.2% | 50.0% |  |
| **Test Set Size** | 946 | 232 | 279 | 176 | 47401 | *N/A* |  |
| **COVID Positive (Test Set)** | 19.0% | 12.1% | 48.0% | 19.3% | 7.6% | *N/A* |  |
| **Feature Type** | Demographics, vitals, Sx | Demographics, vitals, Sx | CT, clinical (sex, age, BT, Sx, exposure, labs) | Sx | Demographics, Sx | Sx |  |
| **Total Features** | 85 | 89 | 12 (clinical) | 15 | 8 | 22 |  |
| **Selected Features** | 26 | 21 | 12 (clinical) | 7 (simplified model) | 5 (biased features excluded) | 14, 22* |  |
| **Leading Features from Best Model** | BT, BMI, SBP, BW, SpO2 | BT, BW, BMI, contact Hx, RR, SpO2 | *Unavailable* | *Unavailable* | cough, fever, contact, male, age 60+ | fever, cough, sudden onset, odynophagia, rhinorrhea (for random forest) |  |
| **Algorithms (Best*)** | random forest*, gradient boosting, extra trees classifier | random forest*, gradient boosting, extra trees classifier | CT: CNN Clinical: SVM, random forest, multilayer perceptron (MLP)* | Lasso regression | gradient boosting (LightGBM) | decision tree, random forest, SVM, NN, & voting ensembles of 2, 3, 4 classifiers (e.g. decison tree + random forest*) |  |
| **AUC (Best algorithm)** | 0.785 | 0.86 | 0.86 (CT), 0.80 (clinical), 0.92 (joint) | 0.783 | 0.86 (“unbiased” model) | 0.728 |  |
| **Accuracy (Best algorithm)** | 0.827 | 0.89 | *Unavailable* | *Unavailable* | 0.921** | 0.681 |  |
| **Sensitivity (Best algorithm)** | 0.339 | 0.29 | 0.836 (CT), 0.806 (clinical), 0.843 (joint) | 0.912 | 0.641** | 0.752 |  |
| **Specificity (Best algorithm)** | 0.941 | 0.98 | 0.759 (CT), 0.683 (clinical), 0.828 (joint) | 0.556 | 0.944** | 0.609 |  |
| **Note** |  |  |  |  | ** A single set of Acc/Sen/Spe was arbitrarily picked |  |  |
|  |  |  |  |  |  |  |  |
|  |  |  |  |  |  |  |  |
